# Mobile applications in the Philippines during the COVID-19 pandemic: systematic search, use case mapping, and quality assessment using the Mobile App Rating Scale (MARS)

**DOI:** 10.1186/s44247-023-00007-2

**Published:** 2023-03-06

**Authors:** Aldren Gonzales, Razel Custodio, Marie Carmela Lapitan, Mary Ann Ladia

**Affiliations:** 1 Medical Informatics Unit, College of Medicine, University of the Philippines Manila, Manila, Philippines; 2 University of the Philippines Manila, 547 Pedro Gil Street, Ermita, Manila, 1000 Philippines; 3 National Telehealth Center, National Institutes of Health, University of the Philippines Manila, Manila, Philippines; 4 Institute of Clinical Epidemiology, National Institutes of Health, University of the Philippines Manila, Manila, Philippines

**Keywords:** Mobile health, COVID-19, MARS

## Abstract

**Background:**

In the Philippines, various mobile health apps were implemented during the COVID-19 pandemic with very little knowledge in terms of their quality. The aims of this paper were 1) to systemically search for mobile apps with COVID-19 pandemic use case that are implemented in the Philippines; 2) to assess the apps using Mobile App Rating Scale (MARS); and 3) to identify the critical points for future improvements of these apps.

**Methods:**

To identify existing mobile applications with COVID-19 pandemic use case employed in the Philippines, Google Play and Apple App Stores were systematically searched. Further search was conducted using the Google Search. Data were extracted from the app web store profile and apps were categorized according to use cases. Mobile apps that met the inclusion criteria were independently assessed and scored by two researchers using the MARS—a 23-item, expert-based rating scale for assessing the quality of mHealth applications.

**Results:**

A total of 27 apps were identified and assessed using MARS. The majority of the apps are designed for managing exposure to COVID-19 and for promoting health monitoring. The overall MARS score of all the apps is 3.62 points (SD 0.7), with a maximum score of 4.7 for an app used for telehealth and a minimum of 2.3 for a COVID-19 health declaration app. The majority (*n* = 19, 70%) of the apps are equal to or exceeded the minimum “acceptable” MARS score of 3.0. Looking at the categories, the apps for raising awareness received the highest MARS score of 4.58 (SD 0.03) while those designed for managing exposure to COVID-19 received the lowest mean score of 3.06 (SD 0.6).

**Conclusions:**

There is a heterogenous quality of mHealth apps implemented during the COVID-19 pandemic in the Philippines. The study also identified areas to better improve the tools. Considering that mHealth is expected to be an integral part of the healthcare system post-pandemic, the results warrant better policies and guidance in the development and implementation to ensure quality across the board and as a result, positively impact health outcomes.

**Supplementary Information:**

The online version contains supplementary material available at 10.1186/s44247-023-00007-2.

## Background

Different countries introduced digital health solutions to strengthen their response to the COVID-19 pandemic. Technologies used range from building information and communication technology infrastructure, aggregating and analyzing data at scale, and enabling virtual and/or artificial intelligence-powered healthcare services [1]. Mobile health or mHealth has been a popular choice because of the high mobile phone penetration around the globe and the recognition of its enormous potential for connecting with health services, surveillance, remote monitoring, and sharing health information [2].

The COVID-19 pandemic has also influenced the growth of mHealth and its rapid adoption [3]. mHealth technologies have been used for early detection, fast screening, patient monitoring, information sharing, education, and treatment management in response to the outbreak [4]. More specifically, mobile health apps address one or more of these use cases: 1) managing exposure to COVID-19; 2) promoting health personal tracking; 3) providing health monitoring; 4) raising awareness; and 5) conducting research [5]. Early pieces of evidence show mHealth’s contribution during the pandemic response. These were in terms of improving public health governance [6], adherence to public health measures, and behavior change [7–9].

Recognizing its potential and growing evidence of its impact, various mHealth solutions were likewise developed and implemented in the Philippines during the COVID-19 pandemic. It was documented that in the first half of 2021, Philippine government-initiated mobile applications implemented to contain the outbreak had two key functions – public awareness measures and health monitoring [2]. Many mHealth tools with health declaration/symptom checker functionalities were also utilized by the national and local government units [10–12]. Health systems and third-party providers launched new apps and tools for telehealth [13–15]. Helplines and chatbots were made available for the public to receive accurate information [16–18].

While having these mHealth tools in the hands of the public is seen as beneficial as the country responds to the pandemic, very little is known in terms of their quality. Recognizing that there are plenty of mobile health apps and it is still expected to increase, there is a need to ensure their quality to make them useful and effective tools. Unlike other countries where policies and standards in mHealth regulation exist, there is none yet in the Philippines – posing risks to current users [19–21].

Recognizing the need to ensure the quality of mobile health apps, the Mobile App Rating Scale (MARS) was developed as a simple and reliable measure. This expert-based rating scale provides a multidimensional approach to assessing mobile health apps to rate both the objective and subjective quality and to identify critical points for future improvements [22]. MARS has been used in a variety of studies assessing mobile health apps. For example, MARS was used to assess the quality of apps for food allergies or intolerances [22], potential drug-drug interaction [23], hematological conditions [24], skin cancer-related [25], orthodontic apps [26], rheumatology [27], HIV/AIDS [28], and Type 2 Diabetes Mellitus [29]. More recently, MARS has been used in other countries to assess COVID-19 apps [30, 31].

The aims of this study were 1) to systemically search for mobile apps with COVID-19 pandemic use cases that are implemented in the Philippines; 2) to assess the apps using Mobile App Rating Scale (MARS); and 3) to identify the critical points for future improvements of these apps.

## Methods

### Search strategy

Multiple search strategies were employed to capture as many mHealth applications for this review. The search was initially conducted using the Apple App Store and Google Play Store (account set in the Philippines) using “COVID-19” as the search term. For the Google Play Store, the search was conducted using a browser instead of using the Play Store app, as the latter showed very few results. While the search resulted in a number of applications, it did not include known applications in the Philippines. Another round of searches was conducted in both app stores using “COVID-19” and “Philippines.” However, initial analysis of the apps showed that it failed to identify many Philippine-specific apps. With this, a Google search was conducted using the terms “COVID-19” and “Philippines” and the first 10 pages were captured, including extracting applications mentioned in online articles (e.g., news, briefs).

### App selection

**Fig. 1:**
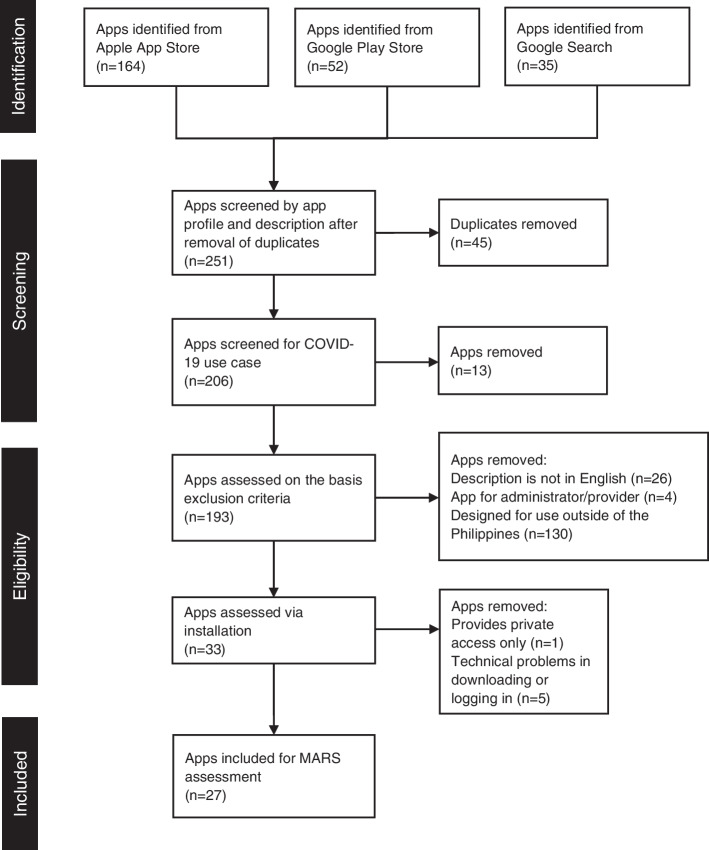
Flow diagram for the selection of the apps

The selection process for apps review is described in Fig. 1. All applications that are implemented for COVID-19 in the Philippines under the following use cases are included: 1) managing exposure to COVID-19; 2) promoting health personal tracking; 3) providing health monitoring; 4) raising awareness; and 5) conducting research [5]. Description and examples of each use case are summarized in Table 1. Apps that are not COVID-19-related, without English translation, system administrator/provider-facing apps, and those designed for specific use outside of the Philippines were excluded. During the assessment, an app is excluded if it still does not work after three attempts.

**Table 1:** Description and examples of each COVID-19 mHealth app use case based from Almalki and Giannicchi [5]

**Managing exposure to COVID-19**: apps guide users to avoid exposure to virus (e.g., apps for contact tracing, reporting suspected cases, granting movement permits, and sharing COVID-19 test results)
**Promoting health personal tracking:** apps help users to look after their health (e.g., apps for self-assessment, symptom trackers, and mood trackers)
**Providing health monitoring**: apps assist users to seek medical assistance from their health care professionals (e.g., apps for virtual medical consultations, making appointments, and helplines)
**Raising awareness:** apps provide various data and information to help users stay informed about this disease (e.g., apps for presenting live statistics and rolling updates, showing the latest news, and chatbots)
**Conducting research study**: apps that would enable researchers to recruit volunteers to take part in COVID-19–related studies and clinical research across countries

### Data extraction

The apps identified in the three searches were listed, counted, and deduplicated. Their descriptions and features were documented during the assessment. Star ratings, number of reviews, developer, and implementer information were captured from the profiles in the online app stores. To categorize the apps, the taxonomy of COVID-19 apps’ purposes by Almalki and Giannicchi was adopted (categories and description in Table 1) [5]. While many of the apps offer multiple features, the apps were categorized following their major functionality.

### MARS app quality assessment

The Mobile App Rating Scale (MARS), a 23-item, expert-based rating scale for assessing the quality of mHealth apps was used to evaluate the quality of the mHealth apps critically and systematically. Published in 2015, the MARS tool has demonstrated excellent internal consistency (alpha = 0.90) and interrater reliability intraclass correlation coefficient (ICC = 0.79). The tool covers five sections that were divided into two categories – the objective and subjective quality. Objective quality has four dimensions (engagement, functionality, aesthetics, and information) with 19 items, while subjective quality is comprised of four items; a total of 23 items. Subjective quality includes questions asking assessors’ intention to recommend the app, frequency of use in the future, willingness to pay, and overall star rating. Each item used a five-point rating scale (1: inadequate; 2: poor; 3: acceptable; 4: good; 5: excellent) [32].

To assess the apps using MARS, the study adopted methodologies utilized by Mandracchia et al. [22] and other publications that used the same tool [23, 24, 26, 31]. First, the research team familiarized themselves with the MARS protocol. They were also trained on the MARS tool through the resource produced by the Institute of Health and Biomedical Innovation at the Queensland University of Technology and the Young and Well Cooperative Research Centre, the developer of the tool. Second, two researchers, both with more than a decade of digital health experience, independently assessed and scored each of the apps. The iOS version was considered first for evaluation and when not available, the android version of the app was used. Lastly, the researchers compared the scores and resolved disagreements together with a third researcher. If an agreement is still not reached, the mean score was calculated as used by Hodges and Sharif [26].

### Statistical analysis

**Fig. 2:**
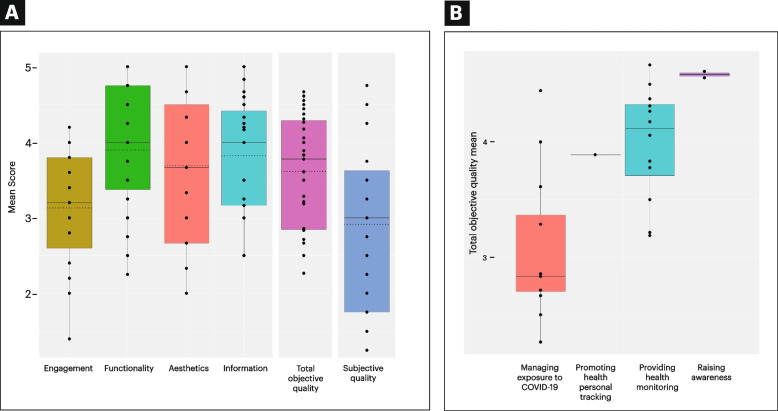
Mobile App Rating Scale (MARS) scores across dimension (**A**) and use case (**B**). Each point represents the score for an individual app (maximum of 5 points). The box plot shows the median (hard line), mean (dotted line), and the first and third quartiles

Quantitative variables were expressed as means and SDs and categorical variables as frequencies and percentages. Following the recommendations of the MARS developers, the MARS score of each app was obtained by calculating the sum of the score of each item divided by the number of total items under the objective sections (sections A-D in Fig. 2). To strengthen the objectivity of the measure, the mean of the subjective quality items was also calculated and analyzed separately.

The pairwise comparisons between the MARS dimensions, use case, developer, and implementer categories were performed using a generalized linear model and a post-hoc Bonferroni test. Because there is only one app under the “Promoting health personal tracking” use case, it was excluded in the comparison. Spearman correlation coefficients described the relationships between the MARS score for each dimension and the user star rating and the number of reviews as used by Kim et al. (2018) [23]. A significance level of *P* ≤ 0.05 was used in this study. The analysis was conducted using R version 4.2.1.

## Results

### Systematic search result

The study identified 251 apps from the Apple App Store, Google Play Store, and Google Search (Fig. 1) conducted in January 2022. After removing duplicates, 206 apps were screened based on inclusion and exclusion criteria. The majority (*n* = 130) of the apps were removed because they were designed for implementation outside of the Philippines. A total of 33 apps were assessed via installation which resulted in excluding six more apps because of technical issues in downloading or logging in and some require private access. A total of 27 apps were included in this study.

### App characteristics

As summarized in Table 2, 17 out of 27 apps were available for both iOS and Android mobile phone operating systems. Private or commercial organizations developed 19 and implemented 13 apps. Twelve apps are designed for managing exposure to COVID-19 while another 12 are those providing health monitoring. Two apps are for raising awareness and only one app for promoting health personal tracking. Individual characteristics of each app and their associated MARS score, number of reviews, and star ratings are included in a supplemental file (see Additional file 1).

**Table 2:** Characteristics of the apps (*n* = 27)

Characteristics of the apps	(*n* = 27)
**Platform available**	
iOS and Android	17
Android only	8
iOS only	2
**Developer**	
Private/commercial	19
Government	4
International organization	4
**Implementer**	
Private/commercial	13
Government	10
International organization	4
**App use case**	
Managing exposure to COVID-19	12
Providing health monitoring	12
Raising awareness	2
Promoting health personal tracking	1

### MARS scores

The overall mean MARS score (considering only the objective dimensions) of the 27 apps assessed is 3.62 points (SD 0.7), with a maximum score of 4.7 for an app used for telehealth and a minimum of 2.3 for a COVID-19 health declaration app. Associating the overall mean score to the MARS scale, the COVID-19-related apps implemented in the Philippines are ranked as “acceptable” (score of 3) to “good” (score of 4). Moreover, 70% (*n* = 19) of apps is equal to or exceeded the minimum acceptable MARS score of 3.0

When comparing the scores of each dimension included (4 objective, 1 subjective), the score for the functionality dimension (mean 3.90, SD 0.8) is found to be significantly higher than the scores of the engagement section (mean 3.13, SD 0.8; *P* = 0.03). The score for subjective quality (mean 3.38, SD 0.2) is also significantly lower than the scores in the aesthetics (mean 3.69, SD 1.0; *P* = 0.02), functionality (mean 3.90, SD 0.8; *P* = 0.001), and information (mean 3.82, SD 0.7; *P* = 0.004) sections. No further significant differences was found in the other between-section comparisons. The scores are plotted in Fig. 2A to visualize the differences between the scores.

### Comparison of MARS scores between the app use case

Looking across the apps grouped according to their use case category (data in Table 3 and total objective score visualized in Fig. 2B), the apps under “Raising awareness” (*n* = 2) received the highest total MARS score (objective) of 4.58 (SD 0.03) while those for “Managing exposure to COVID-19” apps (*n* = 12) received the lowest mean score of 3.06 (SD 0.6). For the subjective score, the mean score of one app under “Promoting health personal tracking” (*n* = 1) received the highest mean of 3.75, followed by the apps under “Providing health monitoring” (*n* = 12) with a mean of 3.69 (SD 1.0). Aside from receiving the lowest objective score, apps for “Managing exposure to COVID-19” also received the lowest subjective mean score (mean 2.00, SD 0.7).

**Table 3:** Differences in the mean MARS scores between app use case

MARS scores	Managing exposure to COVID-19 Mean (SD)	Promoting health personal tracking Mean^a^	Providing health monitoring Mean (SD)	Raising awareness Mean (SD)	All apps Mean (SD)	*P* value^b^	*P* value^c^	*P* value^d^
Engagement	2.55 (0.6)	3.80	3.57 (0.5)	3.70 (0.1)	3.13 (0.8)	0.00079	0.04772	1.00000
Functionality	3.38 (0.8)	3.75	4.27 (0.5)	4.86 (0.2)	3.90 (0.8)	0.0072	0.0170	0.6943
Aesthetics	2.94 (0.9)	3.00	4.28 (0.6)	5.00 (0)	3.69 (1.0)	0.0008	0.0052	0.6765
Information	3.38 (0.6)	4.50	4.03 (0.5)	4.91 (0.1)	3.82 (0.7)	0.0361	0.0061	0.1653
Total objective score	3.06 (0.6)	3.89	3.99 (0.5)	4.58 (0.03)	3.62 (0.7)	0.0015	0.0052	0.5398
MARS Subjective score	2.00 (0.7)	3.75	3.69 (1.0)	3.38 (0.2)	2.92 (1.2)	0.00019	0.13433	1.00000

^a^no SD is provided since only one app falls under this category

^b^Comparison between managing exposure to COVID-19 and providing health monitoring

^c^Comparison between managing exposure to COVID-19 ad raising awareness

^d^Comparison between raising awareness and providing health monitoring

Further assessment showed significant differences in the scores of each app category. For the overall MARS score (objective sections only), apps under “Providing health monitoring” (mean 3.99, SD 0.5) and “Raising awareness” (mean 4.58, SD 0.03) are significantly higher than the scores received by “Managing exposure to COVID-19” (mean 3.06, SD 0.6; *P* = 0.002; *P* = 0.005). Analysis of the four objective dimensions showed that the scores of apps under “Providing health monitoring” and “Raising awareness” are significantly higher than the scores of “Managing exposure to COVID-19.” For the subjective section, the mean score of apps for “Providing health monitoring” (mean 3.69, SD 1.0) is significantly higher than those for “Managing exposure to COVID-19” (mean 2.00, SD 0.7; *P* = 0.0002).

### Comparison of MARS scores between the app developers and implementers

When the total MARS score (objective dimensions only) is compared between developers (Table 4), apps developed by international organizations had the highest with the mean score of 3.71 (SD 1.0) while those from the government scored the lowest (mean 3.17, SD 0.7). The differences in the total MARS score and other section scores, however, were not statistically significant.

**Table 4:** Comparison of MARS scores between app developers

MARS score	Mean (SD) Developer^a^			*P* value^b^	*P* value^c^	*P* value^d^
	**Government**	**Private/ commercial**	**International organization**			
Engagement	2.70 (0.8)	3.22 (0.8)	3.15 (0.7)	0.7	1.0	1.0
Functionality	3.12 (0.9)	4.07 (0.6)	3.88 (1.2)	0.1	0.5	1.00
Aesthetics	2.75 (0.5)	3.99 (1.0)	3.75 (1.5)	0.2	0.5	1.0
Information	3.85 (0.6)	3.88 (0.7)	4.04 (1.0)	1	1	1
Total objective score	3.17 (0.7)	3.69 (0.7)	3.71 (1.0)	0.7	1.0	1.0
Subjective score	2.31 (1.0)	3.14 (1.2)	2.44 (1.1)	0.6	1.0	0.8

^a^Mean of the objective dimensions (engagement, functionality, aesthetics, and information)

^b^Comparison between government and private/commercial

^c^Comparison between government and international organization

^d^Comparison between private/commercial and international organization

For the implementers (Table 5), apps implemented by private or commercial entities had the highest mean score of 3.89 (SD 0.6) while those implemented by the government had the lowest (mean 3.22, SD 0.7). The score of the engagement section was significantly higher in apps implemented by private or commercial entities (mean 3.46, SD 0.6) than those by the government (mean 2.70, SD 0.8; *P* = 0.05). Moreover, apps implemented by private or commercial entities (mean 4.13, SD 0.8) also had a significantly higher score in the aesthetics section than those by the government (mean 3.10, SD 0.9; *P* = 0.05). No other significant difference was found in the other between-implementer comparisons.

**Table 5:** Comparison of MARS scores between app implementers

MARS score	Mean (SD) Implementer^a^			*P* value^b^	*P* value^c^	*P* value^d^
	**Government**	**Private/ commercial**	**International organization**			
Engagement	2.70 (0.8)	3.46 (0.6)	3.15 (0.7)	0.05	0.87	1.0
Functionality	3.50 (0.8)	4.21 (0.6)	3.88 (1.2)	0.1	1.0	1.0
Aesthetics	3.10 (0.9)	4.13 (0.8)	3.75 (1.5)	0.05	0.79	1.0
Information	3.58 (0.8)	3.95 (0.6)	4.04 (1.0)	0.7	0.8	1.0
Total objective score	3.22 (0.7)	3.89 (0.6)	3.71 (1.0)	0.1	0.8	1.0
Subjective score	2.30 (0.8)	3.54 (1.1)	2.44 (1.1)	0.02	1.0	0.22

^a^Mean of the objective section (engagement, functionality, aesthetics, and information)

^b^Comparison between government and private/commercial

^c^Comparison between government and international organization

^d^Comparison between private/commercial and international organization

### Relationship between MARS score and the number of reviews and star ratings

The relationship between the MARS score and the number of reviews and star ratings was assessed using correlations as shown in Table 6. The test showed no significant correlations across the measures.

**Table 6:** Correlation coefficients between MARS scores, user star ratings, and number of reviews

Mobile App Rating Scale	Number of reviews	Star ratings	*P* value^a^	*P* value^b^
Functionality	0.1595767	0.2935907	0.4266	0.1372
Aesthetics	0.2249282	0.322772	0.2593	0.1006
Number of reviews	1	0.03459369	2.2e-16	0.864
Engagement	-0.006598203	0.2322369	0.9754	0.2438
Information	0.01381445	0.3629245	0.6632	0.0628
Total objective quality	0.09850336	0.3737956	0.5778	0.05477
Subjective quality	0.008283561	0.1563657	0.8794	0.4361
Star ratings	0.03459369	1	0.864	2.2e-16

^a^Correlation between MARS scores and number of reviews

^b^Correlation between MARS scores and star ratings

## Discussion

In this study, a systematic search was conducted to identify COVID-19-related mHealth apps implemented in the Philippines. The 27 apps included were mapped to a use case taxonomy and assessed to document their quality using MARS. The majority of the apps were developed and implemented by private or commercial entities. While this is consistent in a study on mHealth apps for persons with multiple sclerosis [33], studies on mHealth apps used in COVID-19 management in other countries showed that the majority of the apps developed were from the government [5, 34]. The result of this study could potentially show the high engagement of the private sector in offering mHealth services during the pandemic as well as the growing business market for mHealth in the Philippines [35].

The majority of the apps identified are designed for managing exposure to COVID-19 (e.g., contact tracing and location monitoring) and providing health monitoring (e.g., virtual consultation and scheduling of services). Although this is not aligned with existing COVID-19 mobile app assessment literature [5, 31], the result could be reflective of the greater need for tools to help users avoid exposure to the virus and for continued access to healthcare services in the Philippine setting.

The assessment of the apps resulted in an overall mean MARS score (objective only) of 3.62 points which could be interpreted as “acceptable” (score of 3) to “good” (score of 4). When compared to the current literature, the overall mean MARS score of this study is lower than those from other COVID-19-related app assessment studies [30, 31, 36] and other domain studies [22, 25, 28]. However, other literature had scores lower than 3.62 points [23, 24, 37–40]. While the result of the study is generally lower than other COVID-19 app assessments, it is also important to recognize that several apps got relatively high scores. For example, a telehealth app for teleconsultation and appointment scheduling received a score of 4.7/5.

Looking at the significant differences in the dimension scores can allow for a better understanding of areas in which the apps are doing well and areas for improvement. Across the apps, the functionality and information dimensions scored the highest. This could be a good indicator as these dimensions are important in ensuring that the app works as designed and that the information is accurate – all are considered important in establishing trust during a pandemic between an app and the user [6, 41]. Lower scores, however, were associated with aesthetics and engagement dimensions. The urgent need to release the apps could have contributed to lower aesthetic quality. Many developers probably prioritized making the functionalities work over aesthetics. This is similar to the results of another study; developers will need to catch up in improving user interface as poorly designed apps can limit the ability of the users to perform their main function [36]. The lower score on engagement could be partially explained by the functionalities and purpose of the apps. Unlike apps designed for behavior change, the apps included in the study do not rely on user engagement [23] and interactivity to achieve its purpose – which could explain the low score in this dimension. For example, users might only be interested in opening a contact tracing app when they need to enter an establishment, or opening a telehealth app if they just need to do a virtual consult.

Although the assessment across apps showed low score on the engagement dimension, the engagement scores are significantly higher for apps implemented by private or commercial entities. One factor that potentially contributed to this finding is that private or commercial entities may have more resources to facilitate enhancements on functionalities that improve engagement of apps with users.

The subjective quality scores are also significantly lower than many of the other dimensions. As discussed in the engagement dimension, the nature and purpose of the apps could have influenced the low rating of this dimension. Especially in the Philippines where the public need to use different applications depending on the location and use case, it is hard to assess when and to whom to recommend the apps and when you would use the same app again. Moreover, while all the apps are free, apps that offer healthcare services (e.g., virtual consult) would require in-app payment – something that is not covered in MARS and resulted in lower scores.

In terms of the use cases, findings show the contrast between the app categories. Significant differences were seen between the total MARS score and dimension scores of apps for managing exposure to COVID-19 and providing health monitoring. Significant differences were also seen between apps for managing exposure to COVID-19 and raising awareness, except for the subjective quality. In both instances, apps for managing exposure to COVID-19 scored significantly lower. Looking at the summary of the scores in Multimedia Annex 1, nine out of 10 lowest-ranking apps fall under this category with the lowest receiving 2.3 points. Aside from the result’s indication of the quality of apps under this use case, the result also highlights that additional effort is needed to improve existing tools. The limited or delays in providing guidance and policies to software developers on contact tracing could have potentially influenced the quality of the apps [42]. Additionally, this result could be a contributing factor or the result of the growing distrust of contact tracing and location monitoring apps among Filipino users – both fall under the managing exposure to COVID-19 category [43].

In looking at the different developers and implementer groups, the study found significant differences in the scores, specifically on engagement and aesthetics, with apps from the government receiving lower scores. The mean score of the apps developed and implemented by government units is lower than the findings in Pakistan [31]. Recognizing that majority of the apps for contract tracing are developed and implemented by the government, the lower quality of these tools could have an implication on how the public perceives the tools and use them. The differences in the quality of apps between sectors highlight the need for the provision and adoption of standards and open an opportunity for cross-sector learning in mHealth design.

Lastly, the study found no significant correlation between the scores and the number of reviews and star ratings. The current literature on this is mixed – some studies found an association between MARS score (total or dimension scores) and the number of reviews and app star ratings [22, 23] while others did not [30]. This could indicate that the number of reviews and the star rating of apps are probably not good indicators of the quality of an app in the Philippines.

### Strengths and limitations

To the best knowledge of the researchers, this is the first study to assess mHealth technologies across the COVID-19 pandemic use cases in the Philippines. Using MARS, a multidimensional and standardized tool, allowed for an objective assessment and comparison of the apps.

However, there are several limitations of the study. First, it is recognized that even with the multiple search strategies implemented, some apps could have been missed. Unlike in scientific publication literature search, no resources were available to researchers in determining the optimal search terms in both app stores. Understanding this will help researchers in conducting systematic searches for apps in the future. Also, some of the apps require registration codes that are unique to targeted users (e.g., employees of an organization), which prevented inclusion in the study. Second, the scores presented only reflect the state of the app during the time of assessment. Like other software applications, mobile apps continue to evolve, and the level of quality could be different with each update/release. This could limit the generalizability of the results. Lastly, the majority of the apps were tested using the iOS app version. While it is common for apps to be developed using a cross-platform environment, other factors would play in the performance of an app (e.g., device specifications, operating system version) when used by different users. This limitation and the non-transparent search algorithms in app stores could challenge the reproducibility of the study.

## Conclusions

Mobile health apps were implemented because of their potential to support public health measures during the pandemic. The systematic search has identified 27 apps, and the assessment revealed differences in terms of quality. While the majority of the apps are above average, others have better opportunities to improve aesthetics, create engagement functionalities, and conforming their apps to quality standards. With the expectation of continuous growth of mHealth apps in the Philippines, guidance and policies (e.g., setting quality standards for mHealth tools) are warranted to ensure their safety and effectiveness to the public. The critical points can help the government, developers, and implementers in understanding areas for improvements in scaling and developing new mHealth tools, especially those supporting the recovery or post-pandemic plans. Establishing better common framework for mHealth assessment could also help developers in ensuring that their tools are aligned and could also help the consumers in selecting the best app for their use. While this study presented a general perspective on the COVID-19 mHealth landscape in the Philippines, future efforts and studies should look at effectiveness of each use case group or individual apps. This could help better understand how the quality of these apps translate to outcome improvements.

## Supplementary Information

**Additional file 1.** Description, characteristics, MARS scores, and ratings of mHealth apps included in the study.

## Data Availability

All data generated or analyzed in this study are included in this published article as a supplemental file. However, the actual names of the apps included were removed. Because of the political landscape and the negative perception of the general public on the tools implemented by the government, the researchers decided not to include the names to avoid any potential misuse or misinterpretation of the data. The goal of the study is to assess the overall landscape of mHealth tools and not draw a conclusion for each app involved.

## Abbreviations

MARS: Mobile App Rating Scale
mHealth: Mobile health
