# Supplementary material for: Mobile applications in the Philippines during the COVID-19 pandemic: systematic search, use case mapping, and quality assessment using the Mobile App Rating Scale (MARS)
Source: BMC Digit Health. 2023 Mar 6;1(1):8. doi: 10.1186/s44247-023-00007-2 (PMC9985954; doi:10.1186/s44247-023-00007-2)
Supplement: Supplementary file 1 — Additional file 1. Description, characteristics, MARS scores, and ratings of mHealth apps included in the study. [file 44247_2023_7_MOESM1_ESM.pdf]

Description, characteristics, MARS scores, and ratings of mHealth apps included in the study

#	App description	Use Case	iOS	Android	Developer	Implementer	Engagement	Functionality	Aesthetics	Information	Total objective quality	MARS app subjective quality	Number of reviews	Star rating
App 1	This app provides health workers with access the COVID-19 knowledge resources including guidance, tools, training, self-paced learning, and virtual workshops to support health workers in caring for patients infected by COVID-19.	Raising awareness	Yes	Yes	International organization	International organization	3.8	5.0	5.0	4.8	4.6	3.3	25	4.6
App 2	A contract tracing app for real-time case management of Local Government Units (LGUs). It also tracks health status and has built-in COVID-19 related information (i.e., stats, usable hotline).	Managing exposure to COVID-10	Yes	Yes	Private	Government	3.2	4.3	5.0	4.0	4.0	3.0	19098	3.6
App 3	A QR Code-based app that automates the contact tracing of agencies under the Aviation and Airports sector of the Department of Transportation (DOTr).	Managing exposure to COVID-10	Yes	Yes	Government	Government	2.0	2.5	2.3	3.3	2.5	1.8	86	1.9
App 4	A case and contact tracing reporting platform for health workers.	Managing exposure to COVID-10	Yes		International organization	International organization	2.8	2.8	2.3	3.2	2.8	1.8	1	5
App 5	An app for mental health and self-care that uses evidence-based screening tools and interventions. It also has an in-app map that locates mental health services available in the Philippines as well as a tab for the list of helplines /crisis hotlines.	Promoting health personal tracking	Yes	Yes	Government	Government	3.8	3.8	3.0	4.5	3.9	3.8	16	3.8
App 6	Enables contact tracing through geolocation; provides users with information on their virus exposure.	Managing exposure to COVID-10		Yes	International organization	International organization	2.4	3.0	2.7	3.2	2.8	1.3	827	3.8
App 7	The app provides telehealth consultation and stores medical records. Users can avail COVID-19 testing through the app.	Providing health monitoring	Yes	Yes	Private	Private	3.4	3.8	4.0	4.0	3.8	3.3	40	3.4
App 8	App for telehealth consults and request for at-home COVID-19 test.	Providing health monitoring	Yes	yes	Private	Private	4.0	4.8	5.0	4.5	4.5	4.8	318	4.4

#	App description	Use Case	iOS	Android	Developer	Implementer	Engagement	Functionality	Aesthetics	Information	Total objective quality	MARS app subjective quality	Number of reviews	Star rating
App 9	A mobile-based platform that facilitates teleconsultation and e-prescription.	Providing health monitoring	Yes	Yes	Private	Private	3.8	4.0	4.3	3.5	3.8	3.3	7	3
App 10	This app provides subscription-based telehealth service via voice call, video call, or chat.	Providing health monitoring	Yes	Yes	Private	Private	4.0	4.5	4.3	4.3	4.3	4.5	3071	3
App 11	A mobile-based platform that facilitates teleconsultation and e-prescription.	Providing health monitoring	Yes	Yes	Private	Private	3.2	4.8	4.3	4.2	4.1	3.3	426	3.6
App 12	A subscription-based telehealth service that connects patients for primary medical care through the aide of nurses, general practitioners, and specialist doctors.	Providing health monitoring		Yes	Private	Private	2.4	3.8	3.7	3.5	3.2	1.8	55	4.7
App 13	An all-in-one app that facilitates 24/7 live video consultation and Telepharmacy. It also has in-app features for healthy recipes, workout plans, and pregnancy guides. It delivers support for COVID-19 patients through assessment, monitoring and test kit appointment	Providing health monitoring	Yes	Yes	Private	Private	4.2	5.0	5.0	4.7	4.7	4.8	10	5
App 14	An app that provides telehealth service via voice call, video call, or chat.	Providing health monitoring	Yes	Yes	Private	Private	3.4	4.0	3.7	3.0	3.5	2.8	169	2.8
App 15	An app that brings all the primary care services such as Teleconsult, e-prescribing, and Telepharmacy.	Providing health monitoring	Yes	Yes	Private	Private	4.0	4.8	4.3	4.3	4.3	4.8	8	3.5
App 16	An app that facilitates teleconsultation, booking of appointments, finding a doctor and, obtaining e-prescription, medical certificate, and laboratory results. It also sends a reminder for COVID vaccine appointments.	Providing health monitoring	Yes	Yes	Private	Private	4.0	4.8	4.7	4.3	4.4	4.5	11	2.2

#	App description	Use Case	iOS	Android	Developer	Implementer	Engagement	Functionality	Aesthetics	Information	Total objective quality	MARS app subjective quality	Number of reviews	Star rating
App 17	A third-party application that links to the Cagayan de Oro City's Health Declaration app. It also generates and scans QR code and allows vaccine registration.	Managing exposure to COVID-10		Yes	Private	Government	1.4	3.0	2.0	3.0	2.3	1.5	35	3.6
App 18	Has in-app health symptoms checker, online library, or knowledgebase for COVID-related information and content management system.	Providing health monitoring	Yes	Yes	Private	Private	3.0	3.3	3.0	3.5	3.2	2.5	41	1.8
App 19	An app that functions as a fully integrated personal medical record management system that integrates with a physician practice management system platform. It also enables users to connect with doctors via telemedicine and schedule clinic appointments in advance.	Providing health monitoring	Yes	Yes	Private	Private	3.4	4.0	5.0	4.6	4.2	4.3	105	3.7
App 20	A logbook app that saves user information for employees and visitors entering or exiting an establishment for contact tracing.	Managing exposure to COVID-10	Yes	Yes	Private	Private	2.2	3.5	2.3	3.0	2.7	1.8	6	3
App 21	A QR-based app for contact tracing and submitting health declaration.	Managing exposure to COVID-10		Yes	Private	Government	3.0	3.8	3.3	3.0	3.3	2.0	3070	4.7
App 22	An app that creates a digital ID with a QR code that would add the Philippine authority to track the citizen travel pass across barangays, municipalities, and cities.	Managing exposure to COVID-10		Yes	Private	Government	2.0	3.8	2.7	2.5	2.7	1.5	92	3
App 23	A contact tracing application that generates an individual QR code and enables the user to scan an establishment's QR code. Additionally, it keeps track of recent locations and includes a vaccine passport feature.	Managing exposure to COVID-10	Yes		Private	Government	2.8	3.0	2.7	3.0	2.9	2.3	16	2.1
App 24	An app that generates travel permits and facilitates travel across the	Managing exposure to COVID-10		Yes	Government	Government	2.8	4.0	3.3	4.2	3.6	2.3	73	2.5

#	App description	Use Case	iOS	Android	Developer	Implementer	Engagement	Functionality	Aesthetics	Information	Total objective quality	MARS app subjective quality	Number of reviews	Star rating
	provinces and cities. It also consists of travel policies and requirements of Local Government Units.													
App 25	A contact tracing app that provides health status notification, health declaration form, QR code identification, and COVID-related news and updates.	Managing exposure to COVID-10		Yes	Private	Government	3.8	4.8	4.3	4.8	4.4	3.5	769	4.2
App 26	An interactive knowledge-transfer platform offering online courses to improve the response to health emergencies, including COVID-19.	Raising awareness	Yes	Yes	International organization	International organization	3.6	4.8	5.0	5.0	4.6	3.5	15	4.3
App 27	A crowdsourcing app that allows commuters to report road conditions and trip tracking. It has also an in-app feature that lets a user request for medical advice or information and to ask for assistance if a user tested positive to COVID-19 or has a medical emergency.	Managing exposure to COVID-10		Yes	Government	Government	2.2	2.3	2.3	3.5	2.7	1.5	6	3.2
